# Instrument for stress-related job analysis for hospital physicians: validation of a short version

**DOI:** 10.1186/1745-6673-8-10

**Published:** 2013-04-17

**Authors:** Monika Keller, Eva Bamberg, Maren Kersten, Albert Nienhaus

**Affiliations:** 1 Department for Work and Organisational Psychology, University of Hamburg, Von-Melle-Park 11, 20146 Hamburg, Germany; 2 Institution for Statutory Accident Insurance and Prevention in the Health and Welfare Services (BGW), Pappelallee 33/35/37, 22089 Hamburg, Germany; 3 Institute for Health Services Research in Dermatology, University Medical Center Hamburg-Eppendorf, Martinistraße 52, 20246 Hamburg, Germany

**Keywords:** Workplace health promotion, Hospital physicians, Stress, Job analysis

## Abstract

**Background:**

Working conditions in hospitals may endanger physicians' health and impair patient care. For this reason, an instrument was developed in the form of a questionnaire, in order to record problems in physicians' working conditions and to suggest possible ways of improving them.

**Methods:**

A survey was performed with 571 hospital physicians. The questionnaire used is a shortened version of the extensive Instrument for Stress-related Job Analysis for Hospital Physicians. This short version contains 14 scales with 30 items on stressors and resources. For validation purposes, several scales were also used for well-being.

**Results:**

The factor structure of the short version of the instrument for hospital physicians was confirmed by confirmatory factor analysis. Cronbach's α and the analyses of interrater agreement with the parameter r_wg(J)_ largely gave moderate to good results. The intercorrelations between the scales are mostly slight to moderate, indicating that the scales are largely independent. The bivariate correlations with different well-being variables are highly significant for most questionnaire scales. In multiple hierarchical regression analyses the scales explained a considerable amount of variance for different well-being variables. Taken together, this emphasizes the relevance of the scales for the stress process.

**Conclusions:**

The short version of the Instrument for Stress-related Job Analysis for Hospital Physicians is a reliable and valid instrument, which can be used practically and economically for normal hospital work.

## Abstract (de)

**German abstract:**

**Hintergrund**

Arbeitsbedingungen in Kliniken bergen für Ärztinnen und Ärzte das Risiko gesundheitlicher Beeinträchtigungen und können sich negativ auf die Patientenversorgung auswirken. Aus diesem Grund wurde ein Instrument in Form eines Fragebogens entwickelt, mit dem Probleme in den ärztlichen Arbeitsbedingungen erfasst und Ansätze für Maßnahmen zur Verbesserung abgeleitet werden können.

Es wurde eine Befragung mit 571 Klinikärztinnen und -ärzten durchgeführt. Bei dem eingesetzten Fragebogen handelt es sich um die gekürzte Fassung des umfangreicheren Instruments zur stressbezogenen Arbeitsanalyse für Klinikärztinnen und -ärzte. Die verwendete Kurzversion beinhaltet 14 Skalen mit 30 Items zu Stressoren und Ressourcen. Zum Zweck der Validierung wurden zusätzlich mehrere Skalen zum Befinden eingesetzt.

**Ergebnisse**

Die Faktorenstruktur der Kurzversion des Instruments für die Klinikärzte wurde mittels konfirmatorischer Faktorenanalyse bestätigt. Cronbachs α sowie die Analysen zur Interrater-übereinstimmung mit dem Kennwert r_wg(J)_ zeigen überwiegend moderate bis gute Ergebnisse. Die Interkorrelationen der Skalen sind zumeist gering bis moderat ausgeprägt und sprechen somit für deren weitgehende Eigenständigkeit. Die bivariaten Korrelationen mit verschiedenen Befindensvariablen sind für das Gros der Fragebogenskalen hochsignifikant, und in multiplen hierarchischen Regressionsanalysen zeigen die Skalen beachtliche Anteile zur Varianzaufklärung des Befindens, was ihre Relevanz im Stressprozess unterstreicht.

**Schlussfolgerungen**

Die Kurzversion des Instruments zur stressbezogenen Arbeitsanalyse für Klinikärztinnen und -ärzte stellt ein reliables und valides Instrument dar, das sich im Klinikalltag praktikabel und ökonomisch einsetzen lässt.

## Background

Hospital work is very stressful for physicians, particularly because of the frequent overtime, shift work and time pressure [1-3]. There are also stressors related to collaboration with colleagues and supervisors, e.g. in the transmission of information [4]. Moreover, in some hospitals, collaboration with supervisors, other physicians and nursing staff is marked by competition and conflicts [2,4-6]. Dealing with patients and their families may also be difficult, due to both emotional and social stress [2,5,7].

Hospital physicians may react to this high level of stress at work with physical and psychological symptoms, such as psychosomatic complaints [7] and emotional exhaustion [4,6,8]. In addition, some studies have concluded that the prevalence of psychiatric diseases is increased [8,9], as is the suicide rate in female physicians [10]. Medical stress is also linked to the quality of patient care, e.g. with medical errors [1].

### Stress-related job analysis for hospital physicians

In order to meet the current difficulties, it is necessary to have a stress-related job analysis, to support hospitals in identifying problems in the physicians' working conditions and in initiating improvements. An instrument of this sort must be capable of recording the specific working conditions of this group, which are characterized by the complex organizational structures in hospitals and the numerous forms of collaboration with supervisors and colleagues from different departments and with other professional groups.

Physicians' work is also characterized by its highly qualified nature and by the enormous responsibility for the patients' life. On the other hand, many young physicians work in hospitals. They are still inexperienced and are highly interested in mastering new methods for examination and treatment, in developing their expertise and in making progress with their specialist training.

Because of the lack of a suitable analytical instrument for hospital physicians' specific working situation, a special questionnaire has been developed for this target group − the Instrument for Stress-related Job Analysis for Hospital Physicians [11,12]. As hospital physicians are normally under great pressure of time, it was important to have a variant of this instrument which could be used economically. For this reason, a short version was constructed, restricted to 30 items.

This article first outlines the development of the short version of the questionnaire (see "Instrument Development"). The study to check its statistical quality is then described.

### Theoretical and methodological principles of the stress-related job analysis for hospital physicians

The questionnaire for hospital physicians is based on transactional model of stress extended for work psychology [13-15]. The central assumption of this model is that both stressors and resources may be *situation*-related or *person*-related. This is equivalent to differentiation on the one hand between *factors initiating stress* and *factors which tend to reduce stress* and, on the other hand, between the *characteristics of the environment* and the *person*. This theoretical framework makes it possible to include in the job analysis stress-relevant characteristics derived from a variety of theories and constructs in work and organizational psychology, e.g. action theory [16].

In the development of the instrument we focused on stressors, such as time pressure and uncertainty, as well as on resources, like autonomy and social support from supervisors and colleagues. Moreover, we concentrated on the situation-based approach for the physicians' questionnaire, because both the results of earlier studies (see above) and of our own preliminary study [17] indicate that most of the physicians' stressors and resources are related to their working conditions and not to their personal characteristics. What is more, this focus on situation-based characteristics provides a good basis for the development of operational interventions.

Methodically, our questionnaire is based on the German *Instrument for Stress-related Job Analysis* (ISTA) [18,19] (descriptions of the instrument are available in Zapf [20] and Semmer, Zapf & Greif [21]), which in turn is founded on the transactional model of stress extended for work psychology, as well as action theory. The ISTA concentrates on situation-based stressors and resources and its items are formulated objectively and specifically, so that their evaluation by the subject is intended to be as independent as possible of their subjective experience. This was adopted for the conception of the instrument for hospital physicians.

## Methods and instruments

### Instrument development

On the basis of extensive preliminary studies, including interviews and shift observations [11,17], a questionnaire was developed to record job-related stressors and resources for hospital physicians. In some cases we adopted original ISTA items for our questionnaire. Due to the fact that many of the ISTA items are largely unsuitable for hospitals, with respect to both the content and the language, we had recourse to other established measures (see below) and several items were reformulated.

The questionnaire was then subjected to a preliminary statistical examination. The preliminary version of the questionnaire included 27 scales with 158 items [11].

This was used in an on-line survey with N=702 hospital physicians. In the course of the statistical evaluations, an exploratory factor analysis was performed first, in order to check the questionnaire structure, which had been adopted for theoretical reasons. Items and scales were then selected, giving a long version of the questionnaire, with 84 items in 23 scales [11]. This was statistically tested during a second survey [12].

Analogously and in parallel to the long version, a short version of the questionnaire was developed. A variety of statistical criteria were used for item selection, including internal consistency, item-scale correlation and face validity. The initial selection of the scales was based on the calculation of bivariate correlations with five different well-being variables. Questionnaire scales were only retained if they gave at least *one* correlation of at least ≥.25 in the overall sample, or two ≥.25 in subsamples (e.g. senior physicians). In a second step, multiple regression analyses were calculated. Scales were retained which explained a significant amount of variance for at least *one* well-being variable [11].

The resulting short version of the questionnaire includes 30 items in 14 scales. The study on the validation of the questionnaire will be presented in this article.

### Short version of the instrument for stress-related job analysis for hospital physicians

**Figure 1:**
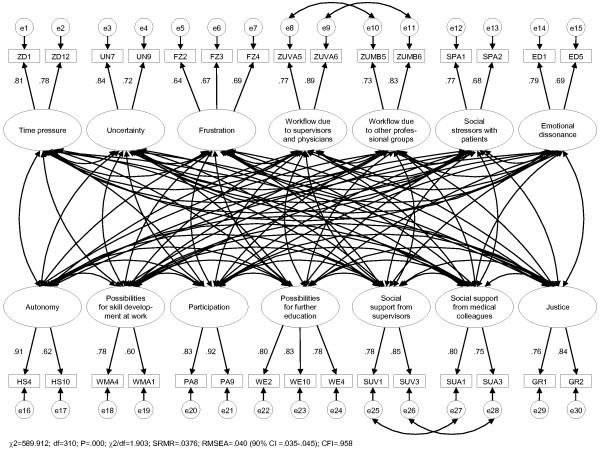
Factor structure of the questionnaire.

The short version of the questionnaire for physicians includes 14 scales, with 2-3 items each (cf. Figure 1). Some of these items are derived from the ISTA scales *time pressure, uncertainty, task control, complexity* and *variability*[18-20]. Most of the items were either reformulated or originate from other established measures (see below).

#### Stressors

7 of the scales focused on stressors. The scale *time pressure* includes two items. One of these items is: "How often are you under time pressure?" *Uncertainty* is also covered by two items. One example is: "How often does it happen that you have to make a decision without having enough information to do this?" The scale *frustration about how work needs to be done* includes three items. One example is: "How often do you not have enough time for patient care, as you have to work on documentation and administration?"

For *problems in workflow due to supervisors and colleagues*, a scale was devised for which the two items were recorded separately for two groups of subjects: (1) physicians (supervisors and colleagues) and (2) employees in other professional groups (i.e. mostly nurses). One example is: "How often does it happen that these persons do not keep to agreements with you?" This means that there is no longer a single scale, but two subscales.

The scale *social stressors with patients and families* also includes two items. One example is: "How often does it happen that patients or their families reproach you?" *Emotional dissonance* is recorded with two (slightly modified) items of the *Frankfurt Emotion Work Scales*[22]. One example is: "How often does it happen during work that you have to suppress your own feelings, in order to give a neutral impression?"

#### Resources

The remaining 7 scales cover resources. The scale *autonomy* includes two items. One example is: "How much freedom do you have to decide for yourself how you perform your work?"

Two scales with two or three items each cover the theme of professional development. One of these scales covers skill development at work. One item is: "How much opportunity do you have to learn new things at work?" The second scale covers possibilities in further education. One item is: "Professional further education for physicians is well supported in our department."

*Participation* is covered by two items. One example is: "I have enough influence on the organization of work in which I am directly involved."

Analogously to the subscales for *problems in workflow due to supervisors and colleagues,* a scale with two subscales was developed for *social support from supervisors and colleagues*. However, the target groups of persons for these scales were defined differently: (1) Supervisors and (2) medical colleagues. The two items for these scales were derived from the scale of Frese [23] (operationalisation according to Caplan [24] and House [25]). One example is: "To what extent can you rely on the following persons when things get difficult at work?"

Finally, two items cover justice. One example is: "Work is fairly distributed among the physicians in our department."

#### Item formats

The items and their alternative answers record specific incidences of work characteristics. One example is: "How frequently do you have to make decision under time pressure?" "Once per month and more rarely" … "Several times per day". Items which cannot be specified in this manner are expressed more abstractly. For example: "How much freedom do you have to decide for yourself how you perform your work?" "very little" …"very much".

All scales have a 5-point response scale and are specified both verbally and numerically.

### Dependent variables

Well-being variables were also recorded as external criteria for the validation of the questionnaire. These were the *irritation scale*[26], the scales for *emotional exhaustion*, *depersonalisation* and *personal accomplishment* in the *Maslach Burnout Inventory* (MBI) [27] and a selection of the items on *psychosomatic complaints*[28].

In addition, a variety of sociodemographic data were ascertained, e.g. gender and professional position.

### Sample and procedure

As the short version of the questionnaire was developed and tested in parallel to the long version, the same sample was used [12]. The study was performed as a paper-and-pencil survey. In all, 1,237 physicians from ten hospitals were surveyed, who had been enrolled with the help of the Institution for Statutory Accident Insurance and Prevention in the Health and Welfare Services.

The participating hospitals were in different regions in Germany, although they were concentrated in the north. Some of the hospitals were in cities and others in the country. The number of physicians employed in each hospital ranged from 12 to 395.

In cooperation with the responsible persons of the hospitals (e.g. staff physician or industrial council), the questionnaire was distributed on site in eight of the hospitals by the study managers, for example, during departmental discussions. However, not all physicians working in the hospitals could be reached in this way, because of lack of time, shift work or leave. The remaining physicians were sent the questionnaire by other routes, e.g. through colleagues or supervisors. It turned out later that the latter routes did not work reliably, so that some of the physicians to be surveyed did not in fact receive a questionnaire. The exact number of undistributed questionnaires can unfortunately not be determined. In three of the hospitals, the questionnaires were collected at a second appointment. Aside from this, the completed questionnaires were sent to Hamburg University, either directly, or through the responsible persons of the hospitals.

### Statistical analyses

Tests were performed on the dimensionality, reliability, interrater agreement and validity of the short version of the questionnaire were calculated. The statistical evaluation used the programs SPSS 15, Amos 16 (for confirmatory factor analysis), IVEware (for the multiple imputation of missing values) and MS Office Excel 2003 (for the calculation of interrater agreement and for handling the results of the imputed data sets).

## Results

### Study participants

571 questionnaires were returned. With a total of 1,237 physicians, this corresponds to a response rate of 46.2%. 57% of the participants in the survey were male. 17% were entrants, with up to 2 years of professional experience; 15% had between 2 and 5 years experience; ca. 20% had between 5 and 10 years experience; 37% had between 10 and 25 years experience: 10% had more than 25 years experience.

The subjects work in various specialities and departments, with marked variations in the number of physicians working in these areas. For example, only 6 pathologists and 12 urologists took part, with 113 physicians working in internal medicine and 95 physicians working in surgery.

### Scale structure of the questionnaire

To test the dimensionality of the questionnaire, a confirmatory factor analysis (CFA) was performed. The model to be tested was specified as follows: (1) All factors (latent variables) are correlated with each other, (2) all items (manifest variables) contribute exclusively to a single factor and (3) the error terms of identically formulated items (for different target groups) are mutually correlated (cf. Figure 1).

The CFA was performed with the maximum likelihood method (ML). In contrast to the assumption in the method, the variables do not show a (multivariate) normal distribution, so that a Bollen-Stine bootstrap correction was also performed [29].

To evaluate the quality of the model, the following fit indices and limits were selected: the standardized root mean square residual (SRMR; <.10), the root mean square error of approximation (RMSEA; <.06) and the comparative fit index (CFI; ≥.95) [29-31]. In addition, chi-square and the normed chi-square (NC; χ^2^/df≤2) were reported [29,32].

The chi-square-statistic is always significant in large samples, so that it is unsuitable as sole criterion for the rejection of a model. However, all other fit indices gave good results: SRMR=0.0376, RMSEA=0.040, CFI=0.958, χ^2^/df=1.903 (Figure 1). This supports the acceptance of the model and indicates that the structure of the questionnaire is valid.

All items exhibited factor loadings of at least .60. They are highly significant and in accordance with the hypotheses. They may therefore be regarded as good [33].

### Reliability

The reliability of the scales was estimated on the basis of the calculated internal consistency or Cronbach's α. Procedures for job analysis use different standards for evaluating the results than are used for analytical procedures in individual diagnostic testing. For the former, α>.60 is regarded as adequate [34], and the item-scale correlation must be r_it_≥.30 [18]. As Cronbach's α overestimates the reliability of scales with only two items, the inter-item correlations were also presented for the affected questionnaire scales.

Table 1 lists the values of Cronbach's α and the range of the item-scale correlations. For scales with only two items, the latter corresponds to inter-item correlations. In any cases Cronbach's α lies above the limit of .60 and must be regarded as being at least adequate. The inter-item correlations are correspondingly somewhat lower. The lowest inter-item correlation is .47 for the scale *Possibilities for skill development at work*. The item-scale correlations for scales with three items are clearly >.30 in all cases and should be regarded as good.

**Table 1:** Means, standard deviations, internal consistency/inter-item correlations (item-scale correlation) and interrater agreement: means* and percentage share in departments with r_**wg(J)**_**values >.50 or >.70**

**Scales**	**M (SD)**	**Cronbach's α/ inter-item correlation (item-scale correlation)**	**Mean r**_**wg(J)**_	**r**_**wg(J)**_**>.50 (in %)**	**r**_**wg(J)**_**>.70 (in %)**
Time pressure	3.62 (.96)	.77/.63	.68	82.7	44.2
Uncertainty	2.59 (1.05)	.75/.61	.63	67.3	36.5
Frustration about how work needs to be done	2.81 (.93)	.71/.48-.56	.64	73.1	42.3
Problems in workflow due to supervisors and physicians	2.15 (.89)	.81/.68	.74	78.8	65.4
Problems in workflow due to other professional groups	2.24 (.82)	.75/.60	.78	98.1	71.2
Social stressors with patients	2.49 (.90)	.68/.52	.69	82.7	48.1
Emotional dissonance	2.53 (1.06)	.70/.54	.43	42.3	9.6
Autonomy	3.36 (.91)	.72/.57	.70	80.8	44.2
Possibilities for skill development at work	3.24(.83)	.63/.47	.74	90.4	61.5
Participation	2.71 (1.02)	.86/.76	.64	75.0	42.3
Possibilities for further education	3.03 (.91)	.84/.68-.74	.79	96.2	76.9
Social support from direct supervisors	3.47 (.96)	.80/.67	.68	75.0	50.0
Social support from medical colleagues	3.73 (.75)	.75/.60	.82	92.3	78.8
Justice	3.12 (.92)	.78/.67	.73	86.5	51.9

N=571; All scales have a 5-point response scale (1-5).

* r_wg(J)_: The arithmetic mean is calculated by transformation of the r_wg(J)_ values in Fisher's Z, taking the mean and back transformation. The results are based on calculations with 52 departments. Values outside the theoretically limits of 0≤r_wg(J)_≤1 were set to zero^44^.

### Interrater agreement

The interrater agreement was determined for all questionnaire scales and for all departments from which at least 5 physicians had participated in the survey. The calculation employed the parameter r_wg(J)_[35], with which the absolute agreement of the physicians within their departments was determined. During the determination of r_wg(J)_, the ratio was calculated of the observed variance within a department to the "randomly expected variance". The latter is what would have been expected if the assessment of the items by the department members had been absolutely random and nothing to do with the real working conditions. It is assumed that the "randomly expected variance" is uniformly distributed [35]. The value of r_wg(J)_ can theoretically lie between 0 and 1, where the value 0 means that there is no agreement of any sort and the value 1 means absolute agreement. The results are evaluated as by James (as in George [36]) and LeBreton and Senter [37]. James gives a limiting value of .70. LeBreton and Senter consider that this value indicates "good agreement" and introduce other standard values. The authors consider that the standard value >.50 indicates "moderate agreement".

r_wg(J)_ was calculated for 52 departments with a total of 517 physicians. Table 1 shows the means and percentage contribution of departments with r_wg(J)_ values of >.50 or >.70. The mean interrater agreement for most scales lay roughly between .60 and .80. According to LeBreton and Senter [37], this must be regarded as moderate to good. An exception is the scale *emotional dissonance*, with a r_wg(J)_ value of .43. The highest mean interrater agreement was found for *social support from medical colleagues*. The proportion of departments with an interrater agreement of >.70 was only above 50% for just under half the scales. On the other hand, the agreement is >.50 for 12 of the 14 scales in at least 70% of the departments.

728 r_wg(J)_ values were calculated, corresponding to 14 scales in each of 52 departments. 28 of these values lay outside the theoretically possible limits (0≤r_wg(J)_≤1). This means that the observed variances in the affected scales and departments were greater than would have been expected if the items had been totally randomly answered. As this only corresponds to 3.9% of the total, it is assumed that this is a chance result. This view is supported by the fact that the values outside the theoretical range of values are distributed over different scales. There is only accumulation in the scales which generally exhibit relatively low interrater agreement: *emotional dissonance*, *frustration about how work needs to be done*, *uncertainty* and *social support from supervisors* (cf. Table 1).

### Construct validity

The intercorrelations between the questionnaire scales were calculated, in order to check the discriminant and convergent validity. According to the hypotheses, there should be positive correlations among the different stressors and among the different resources, but negative correlations between individual stressors and resources. Scales of similar content should exhibit higher coefficients of correlation than scales for which the underlying concepts have no common features.

The signs of all scale intercorrelations (Table 2) are as expected. The stressors exhibited positive correlations with each other and negative correlations with the resources. The resources also correlated positively with each other.

**Table 2:** Intercorrelations of the scales

	**1**	**2**	**3**	**4**	**5**	**6**	**7**	**8**	**9**	**10**	**11**	**12**	**13**
1. Time pressure													
2. Uncertainty	.27**												
3. Frustration	.37**	.34**											
4. Workflow: supervisors and physicians	.18**	.30**	.24**										
5. Workflow: other professional groups	.21**	.22**	.28**	.64**									
6. Social stressors with patients	.18**	.28**	.40**	.14**	.22**								
7. Emotional dissonance	.26**	.30**	.35**	.16**	.23**	.39**							
8. Autonomy	-.12**	-.24**	-.25**	-.24**	-.23**	-.13**	-.20**						
9. Possibilities for skill development	.02	-.01	-.09*	-.17**	-.13**	.06	-.03	.30**					
10. Participation	-.09*	-.21**	-.32**	-.22**	-.20**	-.12**	-.14**	.39**	.36**				
11. Possibilities for further education	-.12**	-.19**	-.28**	-.28**	-.28**	-.09*	-.11*	.26**	.35**	.46**			
12. Social support: supervisors	-.13**	-.24**	-.17**	-.45**	-.29**	-.03	-.10*	.20**	.30**	.31**	.42**		
13. Social support: medical colleagues	-.09*	-.20**	-.12**	-.43**	-.32**	-.11*	-.11**	.17**	.28**	.27**	.33**	.47**	
14. Justice	-.14**	-.17**	-.22**	-.38**	-.29**	-.17**	-.11*	.16**	.23**	.32**	.37**	.39**	.44**

N=571; * p<.05; ** p<.01.

The values of most of the correlations are low to moderate, indicating that the scales are largely independent. Higher intercorrelations were found in the subscales on *social support* and for some resources in the social area or at the departmental level. The highest correlation is .64, which was found for the subscales on *problems in workflow.* An additional CFA was performed to check whether these subscales should be combined to a common scale. The results of this CFA showed that the model fit had considerably deteriorated, indicating that the two subscales should be retained.

### Criterion validity

Criterion validity was determined by bivariate correlations to the five well-being variables recorded. The hypothesis was assumed that the stressors correlate positively with the impairments in well-being and negatively with *personal accomplishment*. The converse relationship is expected for resources.

With a few exceptions, all correlations with the well-being variables are highly significant and (if significant) all in accordance with the hypotheses. The stressors all exhibit positive correlations with the scales *irritation*, *emotional exhaustion*, *depersonalisation* and *psychosomatic complaints* and negative correlations with *personal accomplishment.* The converse correlations were found for the resources (Table 3). In general, the working conditions exhibited the highest correlations with *emotional exhaustion* and the lowest with *personal accomplishment*.

**Table 3:** Bivariate correlations between the stressors and resources and the well-being variables

	**Irritation**	**Emot. exhaustion**	**Depersonalisation**	**Pers. accomplishment**	**Psychosomatic complaints**
Time pressure	.23**	.30**	.19**	.06	.11**
Uncertainty	.27**	.36**	.31**	-.15**	.26**
Frustration about how work needs to be done	.26**	.34**	.24**	.00	.21**
Workflow due to supervisors and physicians	.29**	.26**	.11**	-.15**	.22**
Workflow due to other professional groups	.28**	.27**	.16**	-.15**	.23**
Social stressors with patients	.21**	.30**	.32**	-.13**	.15**
Emotional dissonance	.30**	.41**	.36**	-.12**	.26**
Autonomy	-.19**	-.22**	-.12**	.23**	-.19**
Possibilities for skill development at work	-.16**	-.25**	-.15**	.28**	-.22**
Participation	-.23**	-.31**	-.22**	.24**	-.21**
Possibilities for further education	-.21**	-.28**	-.19**	.24**	-.21**
Social support from direct supervisors	-.28**	-.30**	-.10*	.17**	-.23**
Social support from medical colleagues	-.20**	-.19**	-.14**	.18**	-.16**
Justice	-.24**	-.23**	-.14**	.15**	-.15**

N=571; * p<.05; ** p<.01.

### Multiple regression analyses

Multiple hierarchical regression analyses were also calculated, in order to check the contribution of the working conditions to the explained variance of each of the five well-being variables. In the first step, professional position, professional experience and gender were used as control variables. The second and third steps incorporated resources and stressors.

All the variables taken together explained between 16 and 38% of the variances of the five well-being variables (Table 4). The greatest amount of the variance was explained for *emotional exhaustion*. This applies both to the total model and to the resources and stressors considered separately. Although the resources and stressors explained similar amounts of the variance for *irritation* and *psychosomatic complaints*, the variance for *depersonalisation* is mainly explained by stressors and for *personal accomplishment* mainly by resources.

**Table 4:** Multiple hierarchical regressions on the prediction of the well-being variables from the working conditions

	**Irritation**		**Emotional exhaustion**		**Depersonali-sation**		**Personal accomplishment**		**Psychosomatic complaints**	
**Variables**	β	ΔR^2^	β	ΔR^2^	β	ΔR^2^	β	ΔR^2^	β	ΔR^2^
**Step 1: control variables**		.04**		.06**		.09**		.04**		.03*
Professional position: MA^+^	-.14		.01		.07		.07		.13	
Professional position: MS^+^	-.23**		-.03		.05		.01		.12	
Professional position: SP^+^	-.13		-.03		.02		.08		.08	
Professional experience	-.10		-.15**		-.17**		.11		-.06	
Gender (male)	-.13**		-.08*		.17**		.00		-.06	
**Step 2: resources**		.13**		.16**		.04**		.11**		.09**
Autonomy	-.01		.03		.09*		.11*		-.03	
Possibilities for skill development at work	-.13**		-.22**		-.15**		.20**		-.17**	
Participation	-.06		-.04		-.04		.05		.05	
Possibilities for further education	.03		-.01		-.03		.09		-.01	
Social support from direct supervisors	-.12*		-.14**		.02		.02		-.10	
Social support from medical colleagues	.00		.05		-.05		.02		.04	
Justice	-.05		.00		.01		.00		.02	
**Step 3: stressors**		.09**		.19**		.16**		.05**		.08**
Time pressure	.12**		.17**		.07		.08		.00	
Uncertainty	.09*		.16**		.15**		-.11*		.15**	
Frustration	-.02		-.01		-.02		.19**		.01	
Problems in workflow due to supervisors and physicians	.09		.07		-.03		-.03		.07	
Problems in workflow due to other professional groups	.07		.04		.03		-.05		.08	
Social stressors with patients	.03		.10*		.16**		-.12**		.01	
Emotional dissonance	.17**		.22**		.24**		-.05		.15**	
**Corr. R**^**2**^**total**		**.24**		**.38**		**.27**		**.16**		**.17**

N=549; * p<.05; ** p<.01.

^+^ Dummy variables were formed for the variable *professional position* for three of the four groups of physicians: MA (medical assistances (without completed specialist training)), MS (medical specialists) and SP (senior physicians).

The β-loading factors from the overall model are given, including control variables, resources and stressors.

The following scales should be emphasized as significant predictors: *possibilities for skill development at work*, *social support from supervisors*, *time pressure*, *social stressors with patients* and *emotional dissonance*. The correlation between *autonomy* and *depersonalisation* and the correlation between *frustration about how work needs to be done* and *personal accomplishment* are contrary to the hypotheses.

The control variables of professional experience and gender were also significant predictors of the well-being variables.

## Discussion

This short version of the Instrument for Stress-related Job Analysis for Hospital Physicians is a questionnaire to record the specific stressors and resources in this target group and is correlated with impairment in well-being. It follows that the instrument can be used to improve the planning of work within hospitals, so as to reduce the development of stress. This questionnaire can be used to record problems in working conditions, to deduce possible approaches to achieve improvements and thus to counteract the development of impairment to health.

As the instrument only contains 30 items, the questionnaire can be integrated into everyday hospital work, in spite of the pressure of time. The scales are short, with only 2-3 items, so that a variety of different work characteristics can be measured, despite the restricted size of the questionnaire.

The structure of this theoretically developed questionnaire was confirmed by the analyses performed and it has been shown that all scales permit reliable and valid recording of stress-related work characteristics.

Bearing in mind the low number of items, the internal consistencies must be regarded as favorable. Moreover, the good results for the factor loadings in the CFA emphasize the (statistical) interrelatedness of the items in the scales.

The results for the interrater agreement were mostly moderate. It should however be remembered that the evaluations for the departments included physicians with different workplaces, different experience and different positions. For example, the moderate agreement in the scale *participation* indicates that the physicians within a department may have more or less possibilities of influence, depending on their professional positions.

This is particularly striking with the scale *emotional dissonance*, which only exhibits weak mean interrater agreement [37]. The values for *uncertainty* and *frustration about how work needs to be done* are also relatively low. These scales also depend relatively strongly on the experience of the individual.

Particularly good results were found for the scales on *social support from medical colleagues, possibilities for skill development at work* and *problems in workflow due to other professional groups*. This too is plausible, as the conditions for most physicians in a single department may be similar and less dependent on individual experience.

The correlations with the well-being variables show that the questionnaire scales record stress-related work characteristics and that these are linked to impairments in well-being and personal accomplishment. The level of the bivariate correlations are in accordance with the values between r=.20 and .30, often found in questionnaires on job analysis [21]. The amount of variance explained must also be regarded as favorable. Standard values of between 10 and 20% have been given [21].

The scales *emotional dissonance* and *uncertainty* are particularly good predictors of well-being. Although a variety of other scales also exhibit high bivariate correlations to the well-being variables, some of these scales show no independent significant effects in multiple regression analysis, which is presumably due to the close relationship of their content to that of other scales. For example, *problems in workflow due to supervisors and physicians* make no independent correlation to the variance for well-being, in part due to its intercorrelations of up to -.45 with social resources.

The scale *possibilities for skill development at work* dominated the regression analyses related to professional development. The scale *possibilities for further education* may have had no effect due to shared variance with this scale.

The scale *frustration about how work needs to be done* exhibited, on the one hand, high bivariate correlations with the well-being variables. On the other hand, effects in the regression analyses were either missing or in discord with the hypotheses. This may indicate suppressor effects. One possible explanation might be that successfully mastering difficult and frustrating working conditions (in the context of other stressors) can evoke the impression of a favorable personal accomplishment.

Whereas the bivariate correlations between *autonomy* and *depersonalisation* were negative − in accordance with the hypotheses −, the corresponding regression coefficient was positive. Here too a suppressor effect is conceivable. One possibility might be that *autonomy* accompanied by stressors and/or the lack of other resources offers the possibility to show more depersonalised behavior.

Comparison of the present results with those of the previous analyses with the long version of the questionnaire for physicians [12] shows that there are wide similarities between the two versions. This applies particularly to the internal consistencies, the intercorrelations and the bivariate correlations with well-being. The explained variance of the five well-being variables explained by all questionnaire scales of the short version (including control variables) is between 1 and 5 percentage points less than with the long version.

The results of the interrater agreement are very different. The means for the long version of the instrument are .01 to .13 higher than for the short version and the proportion of departments with r_wg(J)_ >.70 (good interrater agreement) is between 8 and 40 percent points higher for the long version [12].

Some limitations of this study should be noted. The correlations between working conditions and well-being cannot be regarded as causal, as all measurements were taken at the same time. A longitudinal study would be needed to allow conclusions about the predictive validity of the questionnaire. It would be desirable to collect additional data with the sample for the present study.

It must be assumed that the simultaneous presentation and self-assessment of working conditions and well-being variables may evoke methodological effects, expressed as excessive correlations [21,38]. There were clear effects of this sort in a study performed by Zapf [38] on the correlations between stressors (e.g. uncertainty, time pressure) and psychosomatic complaints. Nevertheless, there were still substantial correlations even after the variance shared for methodological reasons had been subtracted.

This study also showed that different work characteristics are influenced to different extents by methodological effects. In contrast to the stressors, no methodological effects were found for the correlations between the variables related to job content (e.g. autonomy and complexity) and well-being [38].

Even though no definitive statements can be made about the results of the present study, it seems reasonable to assume that the results could be influenced in a similar manner to those of Zapf [38].

## Conclusions

The short version of the Instrument for Stress-related Job Analysis for Hospital Physicians, as presented here, is a reliable and valid questionnaire. Because of its shortness, the instrument can be used practicably and economically. The long version is of value for scientific studies.

In spite of its shortness, the questionnaire covers a variety of stressors and resources linked to impairments in well-being. These could provide hospitals with indications about the problems their physicians have in their conditions at work and thus suggest approaches for improvements.

## Competing interests

The authors have no competing interests.

## Authors’ contributions

MoK made substantial contributions to conception and design of the study as well as in the acquisition of data; she was involved in the development of the instrument as well as in the analysis and interpretation of data; she was involved in drafting the manuscript as well as revising it critically. EB made substantial contributions to conception and design of the study, was involved in the development of the instrument and the interpretation of data; she was involved in drafting the manuscript as well as revising it critically. MaK was involved in the development of the instrument and in the acquisition of data, she was involved in drafting the manuscript as well as revising it critically. AN made substantial contributions to conception and design of the study, was involved in the development of the instrument and was involved in drafting the manuscript as well as revising it critically. All authors read and approved the final manuscript.
